# A Smart Biometric Identity Management Framework for Personalised IoT and Cloud Computing-Based Healthcare Services

**DOI:** 10.3390/s21020552

**Published:** 2021-01-14

**Authors:** Farnaz Farid, Mahmoud Elkhodr, Fariza Sabrina, Farhad Ahamed, Ergun Gide

**Affiliations:** 1 School of Computer Science, The University of Sydney, Darlington, NSW 2008, Australia; farnaz.farid@sydney.edu.au; 2 School of Engineering and Technology, Central Queensland University, Sydney, 2000 NSW, Australia; f.sabrina@cqu.edu.au (F.S.); e.gide1@cqu.edu.au (E.G.); 3 School of Computer, Data and Mathematical Sciences, Western Sydney University, Kingswood, NSW 2747, Australia; f.ahamed@westernsydney.edu.au

**Keywords:** identity management, personalized healthcare, authentication, cloud computing, internet of things, fused-based biometric, machine learning, security, privacy, cybersecurity

## Abstract

This paper proposes a novel identity management framework for Internet of Things (IoT) and cloud computing-based personalized healthcare systems. The proposed framework uses multimodal encrypted biometric traits to perform authentication. It employs a combination of centralized and federated identity access techniques along with biometric based continuous authentication. The framework uses a fusion of electrocardiogram (ECG) and photoplethysmogram (PPG) signals when performing authentication. In addition to relying on the unique identification characteristics of the users’ biometric traits, the security of the framework is empowered by the use of Homomorphic Encryption (HE). The use of HE allows patients’ data to stay encrypted when being processed or analyzed in the cloud. Thus, providing not only a fast and reliable authentication mechanism, but also closing the door to many traditional security attacks. The framework’s performance was evaluated and validated using a machine learning (ML) model that tested the framework using a dataset of 25 users in seating positions. Compared to using just ECG or PPG signals, the results of using the proposed fused-based biometric framework showed that it was successful in identifying and authenticating all 25 users with 100% accuracy. Hence, offering some significant improvements to the overall security and privacy of personalized healthcare systems.

## 1. Introduction

The Internet of Things (IoT) and Cloud Computing technologies are shaping and modernising healthcare services. The penetration of IoT devices in the healthcare industry is on the rise. The IoT market in healthcare is expected to reach 135.87 billion dollars of value by the year 2025 [1]. The adoption of Cloud technologies in Clinical Healthcare services is also gaining momentum amongst major players. In the USA, for example, IBM and Aetna developed a “Collaborative Care Solution” to enable easy healthcare access for dispersed Clinical information [2]. Allscripts and MicroHealth are working to provide the US department of state with a cloud-based health solution for its global Electronic Health Records (EHR) management [3]. This solution will provide the healthcare and clinical staff at the department of state with easy access to the patients’ health records. This will benefit both the patients and healthcare professionals [3]. Microsoft is also working with Allscripts on a solution which will enable researchers to conduct studies based on cloud-based EHR [4]. Healthcare providers across Europe and Asia are also working on projects to integrate the IoT and Cloud Computing technologies in their health services [5,6,7].

Nonetheless, most of these works are intended for hospital-based care. While it is still in the development stage [8], Personalised Healthcare (PH) is envisioned to transform the future of healthcare not only in hospitals but also at homes and on the move. PH aims to tailor healthcare services (treatment, medication, diet, and others) to the individual needs of a patient. PH is not only about providing a personalized treatment plan to individual patients but also encompasses services such as earlier prediction and intervention of onset of diseases; and providing a deeper knowledge of the factors contributing to an illness and, hence, offering better-individualised treatment plans.

The Internet of Things (IoT) incorporates multiple long-range and short-range sensors and actuators, RFID tags, smart devices, and personal area wireless networks and technologies (e.g., wearable technologies) into the designs of IoT applications [9]. Combined with the Cloud Computing capabilities of accessing data anytime and anywhere, CloudIoT is leading a digital revolution in healthcare. IoT devices collect diverse data from an individual which provides healthcare professionals with access to a range of complex data, which has never been available before. The data collected from individuals are then connected and mined with data collected from other patients using artificial intelligence and machine-learning techniques. This approach provides insights into the user’s lifestyle, including their diet, physical activities, mental state, genetic composition, environmental factors, amongst many others.

The above-mentioned capabilities paves the way to a new future of personalized healthcare by ways of providing tailored recommendations to patients as individuals or as a cohort. While IoT plays a major role in the collection of this data, Cloud Computing enables such data analysis. Machine learning (ML) is as equally important in PH applications as well. Personalised diabetic management is an example of a PH service that uses the IoT and ML techniques. The system provides dietary advice to an individual based on analyzing their food habits and insulin response using a CloudIoT based application [10].

Although many more promising PH oriented services are emerging, security and privacy concerns remain amongst the major challenges to their fast adoption [11]. More specifically, processing personal data over the Cloud poses many such issues [12]. PH CloudIoT applications rely on the Cloud to store the collected data from the user. The Cloud is also responsible for data computation, processing and analytics. The major challenge in this paradigm is the associated security risks. This includes loss of governance, an increase in threats to data confidentiality due to multi residency threats, the centralization of data on Cloud infrastructure, and the need for privacy solutions that preserve the users’ privacy [13]. Common communication scenarios involve the collection of users’ data using heterogeneous IoT devices and networks. This data will then be sent to the Cloud for further processing ranging from data storage to data analysis and mining. Healthcare workers, thirds parties and other applications could then access the patients’ records and provide personalized health plans. In such a complex, dynamic and ever-expanding environment, obtaining the user’s consent and administering authorization and identity management models are challenging to meet.

Consequently, this paper proposes an identity management framework referred to as the CloudIoTPersonalCare to overcome some of the challenges mentioned above. It integrates the Centralised and Federated Identity Management System (IDMS) for access control. It uses encrypted biometric traits for authentication to ensure security and privacy. The biometric parameters and patient data both are encrypted using Homomorphic Encryption to preserve the confidentiality of the patients’ data. The aforementioned technique has been preferred over other counterparts due to inherent suitability for securing medical data analytics [14].

In this study, the authentication mechanism is achieved using a fusion of electrocardiogram (ECG) and photoplethysmogram (PPG) signals. These two traits are selected since most of the IoT devices in a PH network can read these signals. The use of a single trait-based biometric like ECG for authentication is not secure enough as it is prone to spoofing and can be forged [15,16]. However, fusing these two signals using geometric mean, in addition to Homomorphic Encryption based communication to PH cloud services, will make their use for authentication more secure and hard to falsify or forge [17]. The experimental work shows that our model was successful in authenticating users based on their biometric traits. The results were also validated and compared against other similar works which authenticated the users based on either their ECG or PPG signals [18]. To this end, this work makes the followings contributions:

1. The design of a novel identity management framework for IoT and cloud-based Personalised Healthcare services.
2. The framework incorporates a new biometric-based authentication method. This method uses a fusion of PPG and ECG signals to uniquely identify users. Privacy is preserved using Homomorphic Encryption.
3. The authentication model has been validated and tested using a fusion of ECG and PPG signals and by extracting the instantaneous frequency and spectral entropy features. The outcomes and results of the experiment were evaluated by comparing them with other similar works, which used either ECG or PPG to perform users’ authentication. It is noted that the model successfully identified all 25 users used in the experiment.

## 2. Background and Motivations

Cloud computing and IoT technologies are contributing to healthcare in many areas [2]. Fitness programs, aged care applications, and remote health monitoring systems are a few examples. Ambient assisted living (AAL) and Personalised Healthcare are also emerging areas that make use of CloudIoT technologies to present assistive technologies to vulnerable users. They aim at easing the daily lives of people with disabilities and help in managing chronic medical conditions. Nonetheless, the inherited security and privacy risks from both the IoT and Cloud Computing domains are driving enormous apprehension from the end-users, such as with the case of patients in healthcare applications [19]. As a result, biometric-based authentication models can help mitigating these risks given their unique identification characteristics and their ease of use as they are almost transparent to the user [20]. Due to the delicate quality and dependability of a single biometric trait-based system, the usage of multiple biometric features is gaining momentum [21]. Multi-modal systems incorporate fusion mechanisms, which are diverse and interesting. Based on the type of the application and dataset in use, these techniques can be performed at the data level, image-level (for image processing application), feature level, score level, and at the decision level. Fusion at the image-level requires image registration, which is not always available. Feature-level fusion can reduce information leakage [22,23], but suffers from the incompatibilities of the dimensionality between the source features. Score-level fusion and decision-level fusion avoid these incompatibilities [24,25], but they also suffer from the information leakage problem. It also requires high computation complexity in the matching stage and high storage cost for several templates.

To this end, this work proposes a biometric-based authentication model that employs a data-level fusion of PPG and ECG signals. Therefore, the risk of information leakage, high storage cost and high computation complexity issues are avoided. Biometric information is considered confidential by nature. Hence, the exchange of such sensitive data is strictly regulated. The Health Insurance Portability and Accountability Act, the Australian Privacy Principles Act and the European Data Protection Directive are all examples of some leading bodies, which strictly regulate the exchange of biometric data [26]. Most of these regulations require the use of encryption before exchanging any user’s biometric data. This is why encryption techniques are widely used in most healthcare applications [27]. The traditional symmetric key and public key cryptography algorithms are some examples. For instance, public key models ensure that the receiver must decrypt the data using matching keys prior to performing any computation on the data [14]. This is because, in cloud-based healthcare applications, it is typical for the data to be accessed by a third or external entities [28].

Notably, the newly advanced Homomorphic encryption technique provides the option of performing computation on the encrypted data without decrypting the actual data [14].Thus, facilitating the exchange of data across a range of healthcare and IoT applications and devices without compromising the security and privacy of the system and the users. This work uses Homomorphic encryption to encrypt the authentication process. The authentication model relies on the useful feature of the Homomorphic encryption in maintaining the confidentiality of the biometric template that is used for authenticating patients’ data. This in turn will be transferred and stored securely on the cloud.

## 3. Traditional Cloud-Based IDMS Systems Challenges

Identity Management Systems encompass authentication and authorization mechanisms and combine different technologies to manage users’ identity verification [29]. A typical identity management system consists of three primary entities: the Identity Provider (IDP), Service Provider (SP) and the user or device. The identity provider is responsible for generating the devices’ or users’ identities, maintaining the users’ information and authenticating and authorising the users. The service provider manages resource provisions and services to users or devices. The users and devices are included in the authority and authorisation processes.

**Figure 1:**
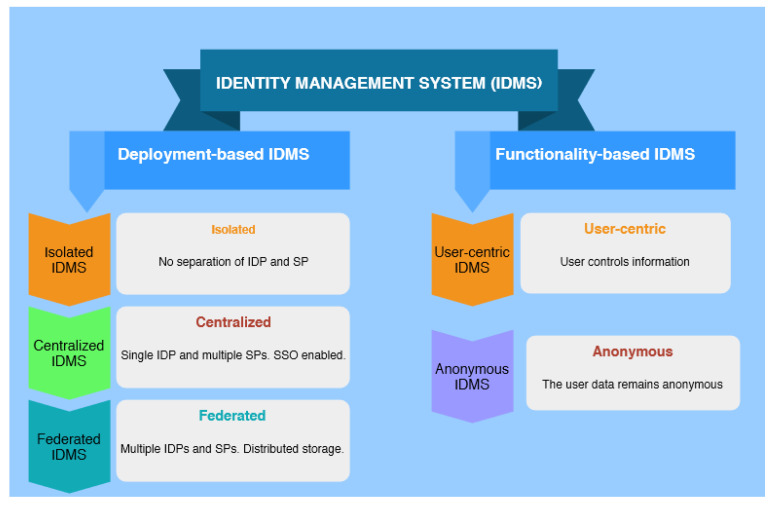
Classifications of an identity management system (IDMS).

An identity management system can be classified into two broad categories: deployment-based systems and functionality-based systems. The deployment-based identity management system is further divided into three categories. These are the Isolated, Centralised and Federated models. Figure 1 depicts this classification.

In the Isolated model, there is no separate service provider. The same party acts as both SP, and IDP [30]. This model is also known as the Silo model since the SPs do not share the users’ identity nor do they share it amongst the SPs. The user may have different identifiers and credentials for each system. These systems do not need to depend on any trusted third party (TTP) for credential issuance and verification purposes. However, users must handle different credentials to access independent systems. The increment in services and resources makes the model hard to manage and cumbersome to the user as users would need to know the credentials of all the services they use [31]. However, this model is more favourable by the service providers given the simplicity of its implementation.

The Centralised model separates the roles and responsibilities of the SPs and IDPs. In this model, there is only one trusted third party, which is a single IDP that is responsible for storing, issuing, and management of identities [32]. The presence of multiple SPs which share the user’s identity enables single sign-on (SSO) systems. The central management Identity Provider (CIDP) collects the identity information. Then, the CIDP sends the authentication request to multiple Service Providers which redirect it to the CIDP. Lastly, a common Identity service provider gets the information from the CloudIoT. If authentication is successful, the service centre offers security to the user. This model is indeed quite beneficial for close environments as it has the usability factor of SSO services. Additionally, both IDP and SP work under the same authority. However, the IDP is a single point of failure and the sole controller of the user identity information. As a result, it might mishandle the information [30].

In the Federated IDMS, the IDP’s functions are shared among several IDPs, which are localised in different security domains. It uses mutual security affirmation and agreement between SPs to allow single sign-on user services [32]. A federation is composed of a trusted group of enterprises of IDPs, and SPs of different domains [33]. The SPs, in this case, accept the authentication token issued by an IDP due to a prior trust relationship established among IDPs and SPs in the federation. The federated model deals with the single point of failure issue suffered by Centralised IDMS. It also eases the user burdens as it removes the need to have to authenticate every time. The users do not have to manage a myriad of identities as well [30]. In these type of systems, the same identification information is used to access all networks within the specific trusted group of enterprises. It is a realisation of a federated identity management model that has the power to enable this process in cloud computing, and IoT environments [34].

Moreover, while using these systems, there is no need to create any other account for external parties. It relies on its inherent cross-domain access. This process is commonly used in the Information Technology industry [35]. In this system, the identity information is stored at multiple locations, given its distributed architecture. Additionally, to increase security, a federated Identity Management Systems and cloud service users request results in the linking of their information across multiple Identity Service Providers [36].

User-Centric Identity Management Systems are used on the user’s end to accumulate, achieve, and recover the user’s information from any unauthorised person. It is also used to exchange their identity credentials with other trusted entities like cloud service providers, identity providers, or other cloud service users [37]. So, the system puts the users in control of their security. Moreover, identity management systems do not disclose identifiable information to the service provider without the user’s consent.

Anonymous Identity Management Systems have a feature of secrecy. An anonymous cloud identity management system can keep the secret of its entity from attackers [38]. In identity management systems, the data might be interconnected with other data. So, it is prone to identity attacks [30]. On the other hand, user-centric IDM models are recommended in e-health applications as they provide the user with better control over their information [39]. However, in some cases, distinct users (e.g., patients, health professionals) which can be localised in different security domains, may need access to a patient’s health data. In this case, the user may not use the same IDP for authentication, therefore rendering the federated model more adequate to use [39].

The preceding discussion demonstrates that no single IDMS is suitable for a diverse and expandable cloud-based system. As a result, in our architecture, we adopt the notion of multi-modal IDMS, which comprises both Centralised and Federated access management models.

## 4. Cloud IoT Personal Care IDMS Framework

**Figure 2:**
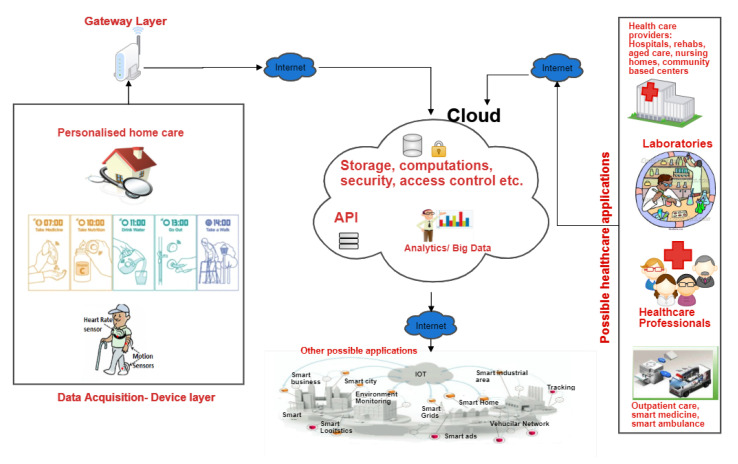
Architecture of CloudIoT Personalised Healthcare (PH) services.

Incorporating Personalised healthcare systems with other IoT systems and cloud technologies opens the door to numerous opportunities. Figure 2 presents some of the possible smart applications that can result from the integration of PH in the IoT.

At the device layer where data acquisition occurs, IoT sensors, wearable devices and other smart devices collect data from patients and send them to the cloud database through the IoT gateway (the Gateway layer). The Gateway layer consists of a range of networking devices which have access to the Internet. These devices form a local cloud on their own to run the authentication computations. They perform the encryption on the biometric template and save it for authenticating the patient later. The Gateway layer also sends the encrypted template to the hospital cloud to ensure correct authorization is done in the healthcare database. This gives rises to several applications that can benefit from accessing this data. It enables healthcare professionals to access the data remotely and empowers other healthcare applications with the capabilities of providing intelligent services such as smart medicine management, emergency alert systems, and community-based engagement services amongst many others. The opportunities become vast when other IoT applications are also incorporated into the cloud IoT system. Obviously, such an interconnected complex system gives rise to numerous challenges in terms of interoperability, security, and privacy, which are out of the scope of this paper. Our work focuses on facilitating the provision of a unique, automated and secure authentication scheme for the data acquired from the users. It achieves this by using the biometric traits of a user as a base for the authentication scheme.

**Figure 3:**
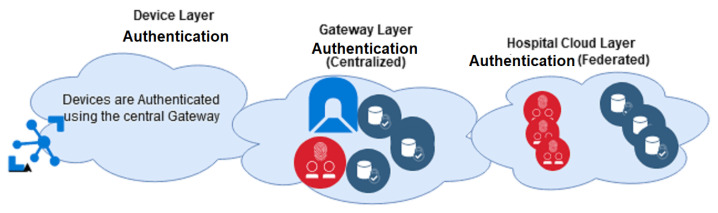
The architecture for the proposed IDMS for PH.

The proposed CloudIoT based healthcare system has three layers: the Device layer, Gateway layer, and the Hospital/Public healthcare cloud layer. Figure 3 illustrates these layers. The Device layer is in the patient’s network where the devices are authenticated using a central gateway. The Gateway layer is where the patient’s authentication credentials are stored and where encryption and any other initial computation are done. The Gateway layer maintains a Centralised access control that facilitates a user-centric restriction on sharing data with the public healthcare cloud. The last layer is the Hospital cloud layer which resides on the public cloud and adopts a Federated IDMS strategy.

### The Authentication Framework Details

**Figure 4:**
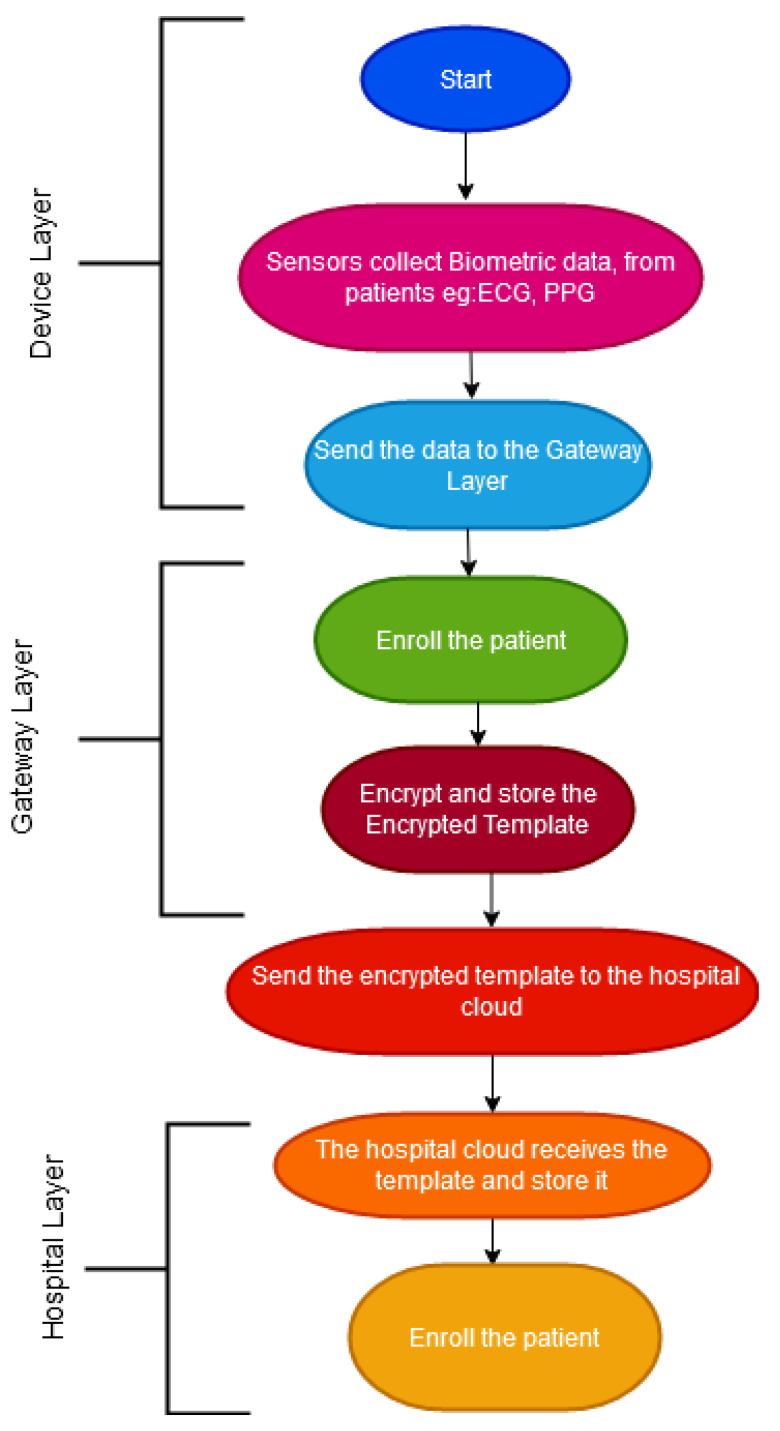
The enrolment phase.

**Figure 5:**
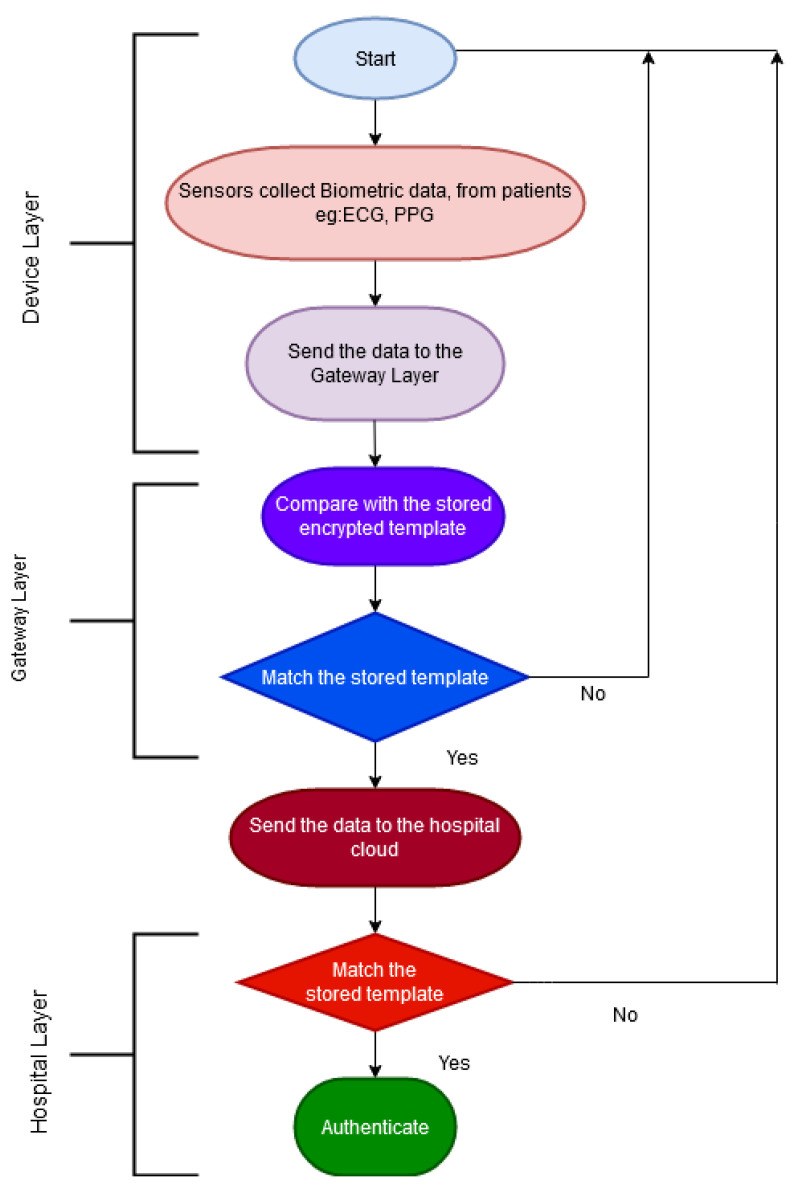
The authentication phase.

The authentication processes involved in the gateway and hospital cloud layer are done in two phases: the enrolment and authentication phases which are shown in Figure 4 and Figure 5, respectively.

- Device Layer: The sensors collect the biometric data at various times from the patients under different settings, such as in a sitting position or when lying down and send it to the gateway layer.
- Gateway Layer: The gateway layer enrols the patient and saves the template. The biometric template is secured using the Homomorphic algorithm. The encrypted template is then sent to the hospital cloud.
- Hospital Layer: This layer stores the authentication data received from the Gateway layer to authorise the patient in the future. This layer is also responsible for computing and analysing the patient’s data.

The steps involved in the authentication process can be summarised as follows:

- The patient sends new data and their biometric traits to the gateway layer through the device gateway.
- The Gateway layer tests the encrypted biometric template against the stored encrypted template in the database. If the template matches, the user is then authenticated.
- The Gateway layer sends the data to the hospital cloud. The user is then authenticated against the stored encrypted biometric template versus the newly arrived template received from the gateway. If the template matches, the user is fully authenticated.

## 5. Encryption for Biometric Template

The proposed authentication framework uses the Homomorphic encryption (HE) to secure patient data. The biometric template and patient data are all encrypted to prevent them from being stolen and misused. One of the advantages of the HE method is that computation can be directly performed on the encrypted data without the need to access the secret key. The result of the calculation stays in an encrypted form as well. Thus, the computation can be done on the ciphertext without decrypting the plaintext [40]. For instance, for a plaintext *PT_i_* if the corresponding ciphertext is *CT_i_* and for *PT_j_*, the corresponding ciphertext is *CT_j_*, the Homomorphic encryption can perform computation on *CT_i_* and *CT_j_* without revealing *PT_i_* and *PT_j_*. The computation is based on the addition or multiplication process. If *HEA*(x) is a function to encrypt the plaintext *PT_i_* and *PT_j_*, the Homomorphic encryption for these texts are computed as follows:

(1) $$CT_{i}=HEA{\left(PT\right)}_{ii}$$

(2) $$CT_{j}=HEA{\left(PT\right)}_{jj}$$

(3) $$CT_{i}\times CT_{j}=HEA{\left(PT+PT\right)}_{ijij}$$

The detailed reasoning behind using this cryptographic primitive have been discussed in Section 2. Table 1 provides the notations used in the equations.

Based on the above concepts, the enrolment level encryption uses the following steps to encrypt the stored biometric template:

- Key tuple generation: In the enrolment phase, the sensor sends the collected biometric template. The template is secured using a key pair secret key $sk_{i}$ and public key $pk_{i}$. The key is generated using the KGen(κ) function, which takes the biometric trait *ECG*/PPG as input to generate a key.
  (4) $$KGen\left(\kappa_{ECG}\right)=pk_{i},sk_{i}$$

However, when the patient sends real health data after the enrolment phase, a new key is generated, combining the patient data and biometric traits such as *ECG* or PPG or a combination of both of them.

(5) $$KGen\left(\kappa_{BT,Data}\right)=pk_{j},sk_{j}$$

where *BT* = biomeric traits, *data* = health data.

- Encryption technique: The biometric template is then encrypted using the encryption function:
  (6) $$C_{BioTemp}=Enc\left(pk_{i},P_{BioTemp}\right)$$

This function takes a public key *pk_i_* and biometric template plaintext *P_BioTemp_* as inputs and outputs a ciphertext which is the encrypted biometric template *C_BioTemp_*.

- Decryption technique: The template is decrypted using the decryption function as follows:
  (7) $$P_{BioTemp}=Dec\left(sk_{i},C_{BioTemp}\right)$$

This function takes a secret key *sk_i_* and encrypt the biometric template ciphertext *C_BioTemp_* as inputs, and outputs the related plaintext *P_BioTemp_*.

- Evaluation function: The next function is the evaluation function Ev(pk,χ,σ). The evaluation function takes the generated public key from the biometric traits, circuit χ with m inputs, where m is the plaintext for biometric template *P_BioTemp_* and a set of *C_BioTemp_* of generated ciphertext from plaintext *P_BioTemp_* which is $C_{BioTemp_{1}}$, $C_{BioTemp_{2}}$, …, $C_{BioTemp_{m}}$ and output a ciphertext *C_BioTemp_*. It works in such a way that χ($P_{BioTemp}$${}_{1}$, $P_{BioTemp}$${}_{2}$, …, $P_{BioTemp}$${}_{m}$) = Dec (sk${}_{i}$, $C_{BioTemp}$) where $\chi_{\Pi }$ is an allowed circuit set of biometric templates and if the ciphertext $C_{BioTemp}$ is the corresponding ciphertext for plaintext $P_{BioTemp}$${}_{i}$ for *i* = 1, 2, …, m and $C_{BioTemp}$ = ($C_{BioTemp}$${}_{1}$, $C_{BioTemp}$${}_{2}$, …, $C_{BioTemp}$${}_{m}$) then the evaluate function Ev(pk${}_{i}$,χ, $C_{BioTemp}$) returns a ciphertext $C_{BioTemp}$ corresponding to the plaintext χ ($P_{BioTemp}$${}_{1}$, …, $P_{BioTemp}$${}_{m}$) for a circuit χ with m inputs.

The patient data is also encrypted using the HE technique. However, the details of the encryption processes are out of the scope of this paper.

### The Adversary Model

The designed encrypted authentication framework is open to two broad categories of adversaries. They are sample recovery and reference recovery attacks [41].

1. Sample recovery attacks: In this type of attack, the perpetrator uses the spoofing or brute force technique. Consider, for example, a rogue sensor that enters the patient’s home network. To authenticate itself as a valid user, it generates an ECG and a key pair. The authentication servers, in this case the gateway layer, will enrol and will list it as a valid sensor and encrypt the template. Now the attacker will break into the real sensor and collect any data the user was sending. The attacker will directly send this data along with the ECG signal. Another key will be generated using the health data of the real patient and ECG of the impersonator. However, the encryption key will differ since the access key of the actual user is distinct. So the sample recovery attack will fail in this authentication scenario.
2. Reference recovery attacks: In this case, an adversary gains access to a patient’s reference biometric traits. As in the previous scenario, the attacker also gets access to the patient’s health data. This time when the data are sent together with the real ECG signal (in the form of replay attack), the gateway layer will be tricked into providing access to data stored at the hospital cloud. To mitigate this type of attacks, in our authentication framework, we are using a signal level fusion which combines ECG and PPG signals and generates a key using these fused signals. It would be nearly impossible for the adversary to spoof both ECG and PPG of the same person.
3. Concomitantly, these type of attacks can happen in the hospital cloud layer. An adversary gains access to the saved encrypted biometric template in the hospital cloud. The rogue service provider RhospCp_i_ will try to access the data. However, data are encrypted using the Homomorphic approach, the RhospCp_i_ will not be able to access the plaintext data and will only be able to perform computation on the data such as send it back to the patient p_i_. To mitigate reference recovery attacks, replay attacks and man in the middle attacks, security measures such as the use of public-key cryptography, TSL/SSL, authorised certificate authority and other classical security measures such as the use of a VPN can be incorporated in the authentication model.

## 6. The Experimental Work

In this section, the experimental work used to validate the proposed authentication model is described. The biometric authentication experiment utilised data sourced from a publicly available dataset [42]. The proposed biometric-based authentication framework is trained and tested using this dataset. The dataset contains ECG and PPG data that were collected from 25 users who participated in an experiment. The participants wore a prototype device in the form of a smartwatch which was placed on the users’ wrist. The prototype device was equipped with an ECG and a low-cost open hardware PPG sensors along with some other sensors such as the Galvanic Skin Response (GSR) sensors. The Physiological data, i.e., the ECG and PPG signals, were collected from the 25 participants in seating postures. The dataset contained data collected from each participant over the duration of five minutes. Each participant produced approximately a sequence of 28,000 samples of amplitude values of PPG and ECG signals.

To confirm the validity and robustness of the authentication model, this experiment was conducted in two phases. In the first phase, we trained and tested the authentication model using the data collected from 10 participants. In the second phase, and in order to test and evaluate the scalability of the experiment, we increased the dataset size to 25 users. The details of these experiments are provided in the subsections below.

### 6.1. Dataset Pre-Processing

Firstly, fusion signals were computed and collected from the users in sitting positions. Using the PPG and ECG signals, a fused signal $F_{s}$ was derived using the following formula:

(8) $$F_{s}=\sqrt{P_{s}^{2}+E_{s}^{2}}$$

**Figure 6:**
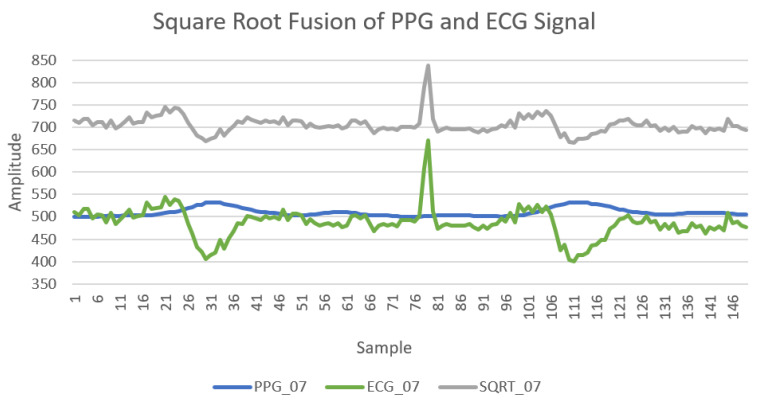
Photoplethysmogram (PPG), electrocardiogram (ECG) and fusion signal.

In Equation (8), the $P_{s}$ represents the PPG amplitude point and $E_{s}$ represents the ECG amplitude point both at a given time. In preparation of phases 1 and 2 of this experiment, the fused signal as well as the PPG and ECG signals were stored in a separate data file (“mergedTable1.mat”). This and the relevant data processing code are published on GitHub [43]. It is worth noting that during the training phase of the model, the minor class (valid user) parameters were duplicated in order to avoid creating an imbalance of valid user samples. A comparative graph of a participant’s PPG and ECG signals along with the resultant fused signal is provided in Figure 6.

Before training the model, the sample sequences of data were normalised first followed by the process of features’ extraction.

### 6.2. Features Extraction

The fused signal represents a sequence of amplitude values. Hence, we extracted two distinct features from the non-stationary signals including (1) the instantaneous frequency and (2) the spectral entropy. The instantaneous frequency feature was particularly helpful for discovering the frequency noise and phase noise. It was computed as the first contingent spectral moment of the time-frequency distribution of the input signal. It can be calculated using the following Equations (9) [44]. Ps(t,f) represents the power spectrum.

(9) $$f_{ins}\left(t\right)\;=\;\frac{\int_{0}^{\infty }f(Ps(t,f))\;df}{\int_{0}^{\infty }Ps(t,f)\;df}$$

The spectral entropy is the Shannon entropy properly normalised and applied to the power spectrum density of the fusion signal. This entropy was also used as part of the features selection for training and testing the model. The spectral entropy (SE) was worked out using Equation (10) [45]: Here, Pd(n) represents a probability distribution, and *N* represents the number of frequency points

(10) $$SE=\sum_{n-1}^{N}Pd\left(n\right)log_{2}Pd\left(n\right)$$

The feature matrix was created after calculating the instantaneous frequency and spectral entropy from the sample signals. These features are provisioned in the input layer of the biometric authentication framework.

### 6.3. Phase One of the Experiment

**Figure 7:**
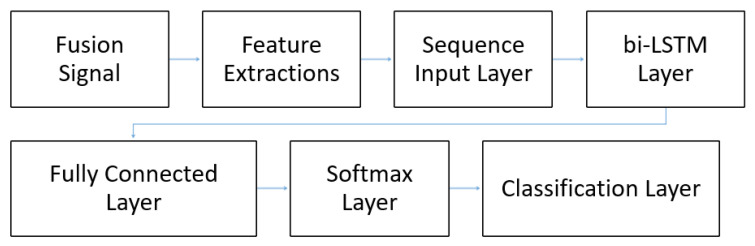
The biometric model architecture with PPG and ECG fusion input.

To perform biometric authentication, we employed the Long Short-Term Memory (LSTM), which is a sequence pattern that matches deep neural networks. This learning algorithm is broadly used for identification and prediction of time series data. We exploited the bidirectional LSTM layer, to enforce the LSTM net to examine the sequence of the ECG and PPG signals in the forward direction as well as in the backward directions to match a valid user. The sample window size for each sequence was set to 1500 for each participant. Each user produced around 18 sequences of samples. Hence, the total sample size produced by 10 users was over 182 sequences of fused samples. The minor class data were also duplicated a few times to improve the training bias of the model. Figure 7 shows the network model architecture.

To find out the number of optimal hidden layers required for this model, we defined the LSTM layer with a variable number of hidden neurons. That is, the fewer the neuron number was, the lower the computation cost was. This layer related to the fully connected layer. There were only two classes of deserving output: a valid or an invalid user. Hence, we included a fully connected layer of two neurons to specify two classes, followed by a softmax layer which distributed the probabilities of each class followed by a classification layer. The softmax layer also served as a neural transfer function. These transfer functions calculated the softmax layer’s output from its net input.

#### The Model Training Setup

**Figure 8:**
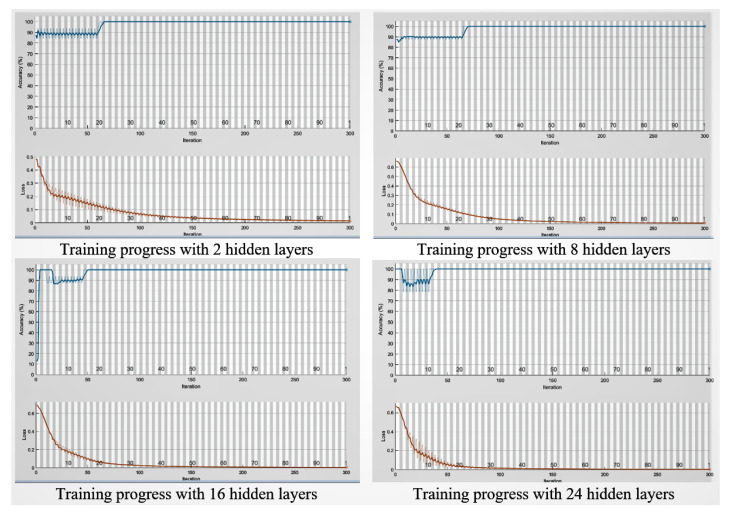
Training progress with various hidden layers.

There were several training options for the classifier. We set some them as follows: The maximum epoch was set to 100 to train the network and configure the neurons. A mini-batch size of 32 was configured that directed the network to consider 32 data points at a time. The initial learning rate was set to 0.01 which quickened the training process. To restrict the gradients from getting too large and to stabilise the training process, the gradient threshold was set to 1. We also used the Stochastic Gradient Descent with the Momentum (SGDM) optimiser. The training progress charts are provided in Figure 8.

The model network received two computed features from Equations (9) and (10). Then, the features went through the BiLSTM layer which had various sets to 2, 8, 16 and 24 neurons—this was in order to compare the network’s performance.

### 6.4. Phase Two of the Experiment

In phase two of the experiment, we loaded the fused signal from the imported file “mergedTable1.mat” into the ML model. In this phase, the ML model had a similar architecture to the one depicted in Figure 7. Using a sample frequency of 150 Hz, each user had 182 sequences of samples. Hence, the total sample size of data corresponding to the 25 users was over 4700 sequences of fused signals. These sequences of signals were used to extract the features using Equations (9) and (10). Next, the features extracted by the equations were fed into the sequence input layer to train the ML model as illustrated in Figure 7.

#### Model Training Options

In phase two of the experiment, 100 neurons were used in the hidden layer of the model. The maximum epoch was set to 20 and the batch size was set to 32 for the training. The initial learn rate was set to 0.01 and the gradient threshold to 1. In multiple trials, one of the users was selected as a valid user to test against the biometric data of the remaining users. Then, 70% of the data were allocated for training the model and the remaining 30% of data were allocated to test the model. Therefore, 190 samples of valid-user data and 4500 samples of attacker signals data were used to train and test the model.

## 7. Results and Analysis

The pre-processed dataset was split into a 70/30 ratio for training and testing to facilitate the experiment. The model was trained to distinguish the fused signal of a valid user from that of other users. After the training phase, the model was then tested with the test portion of the data. The model’s performance in recognising valid users was then noted and validated.

### 7.1. Results: Phase 1

The results of phase 1 of the experiment are presented in Table 2 and Table 3. TN stands for True Negative, TP stands for True Positive, FN stands for False Negative and FP stands for False Positive values. From Table 2 and Table 3, True Negative (TN) means an attack is identified as successful. True Positive (TP) means that the valid-user has been successfully authenticated. Table 2 and Table 3 exhibit that, the training and test model could reach maximum precision and accuracy of 100 per cent by using only two hidden layers of LSTM. Therefore, we note that a smaller LSTM network is considered to be suitable for biometric authentication validation purposes.

In phase 1 of the experiment, we also sampled data with a sequence size of 15 s which corresponded to 1500 amplitude points. The results demonstrated that 15 s of fused ECG and PPG signals were adequate for the model to authenticate a user.

Table 2 and Table 3 show that the proposed biometric authentication model could achieve a 100% accuracy with just two hidden layers. The model became optimal after 100 epochs. We have compared the model’s performance in terms of accuracy and equal error rate (EER) with other similar works [42].Thus, under the same settings i.e., in sitting postures and using the same data source, our model which fused ECG and PPG signals to authenticate users outperformed the performance of the model reported in [42], which used three physiological signals including the ECG, PPG, and GSR. The results are reported in Table 4. When using only the PPG signal for authentication, the success rate of the model in authenticating the 10 subjects achieved 100% accuracy [18]. The experiment results demonstrated that the proposed model also achieved an accuracy rate of 100%. However, given that our model relied on a fused ECG and PPG signal, it was considered more secure and less vulnerable to spoofing attacks. It is more difficult for an attacker to forge both ECG and PPG signals, fuse them, and calibrate them when compared to just spoofing either the ECG or PPG signal.

### 7.2. Results of Phase 2 of the Experiment

**Figure 9:**
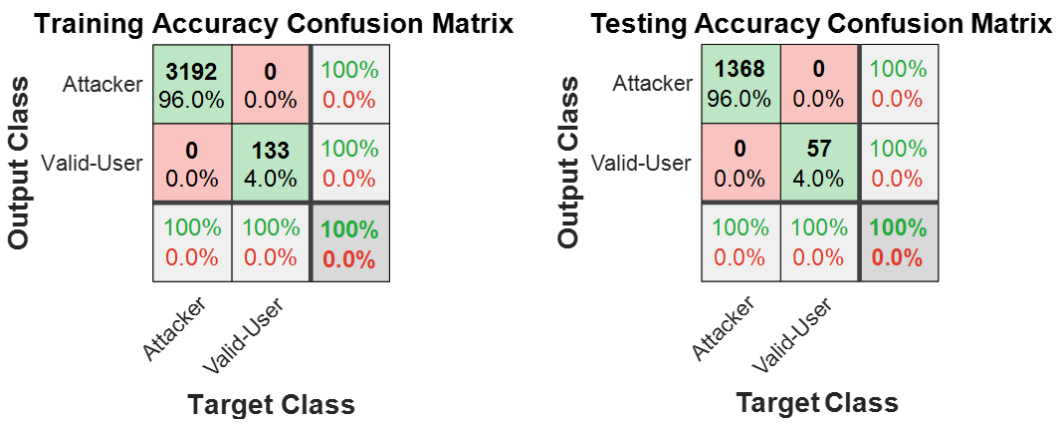
Phase two experiment confusion matrix.

In phase two of the experiment, the dataset sample size was increased from 10 to 25 users. This allowed us to further test and confirm the performance of the proposed model. The results are presented in Figure 9 as a confusion matrix. Figure 9 shows that the performance of the training and testing setups both achieved 100% accuracy. Although the experiment was set up with a 70 to 30 train and test ratio, we discovered that a smaller sample size was also sufficient to train the model in recognising valid users—even after reducing the train/test ratio to 20/80.

Based on the reported outcomes of the two phases of the experiment conducted in this work, it is evident that a fused signal of ECG and PPG can be used to authenticate users based on their biometric traits without compromising the accuracy of the model. Compared to using just ECG or PPG signals, the outcomes of using the proposed fused-based biometric model showed significant improvement to the security and privacy of the overall personalized healthcare system.

## 8. Limitations

This work acknowledges a few limitations. The proposed biometric-based authentication model was tested to be secure against spoofing attacks. End-to-end security has not been validated. Thus, classical identity-based security attacks, such as man in the middle and replay attacks, are yet to be tested. As many of these attacks can be mitigated using existing established security measures such as the use of VPN, validating the end to end security aspects of the proposed model is planned in future work. Nevertheless, the use of Homomorphic Encryption by the proposed model allows data to stay encrypted when being processed or analysed in the cloud or when accessed by third-party devices, thus reducing the risk of data theft and leakage.

Lastly, the dataset used to conduct the experiment sourced the PPG and ECG signals from users in sitting postures. Future work will look into evaluating the performance of the proposed model in authenticating users while they are in various positions e.g., walking or climbing stairs.

## 9. Conclusions

This work proposed a biometric identity management framework. The framework is based on multi-modal IDMS and the Homomorphic Encryption technique. It provisions the unique identification of patients’ identities in personalized healthcare environments. The authentication process is based on recognisable and verifiable biometric data. It uses a novel approach that fuses ECG and PPG signals when performing authentication, thus providing a reliable, fast, and most importantly, a secure authentication method. The proposed approach alleviates many of the issues encountered in the health domain as most elderly patients usually lack the experience of using technologies. The approach of using a fused-based biometric approach mitigates many of the security risks associated with the use of a single biometric trait (e.g., PPG signals), such as spoofing attacks. The experimental works reported in this paper also confirm the success of the framework in identifying users based on their biometric profile, mainly based on fusing their ECG and PPG signals. The performance of the proposed biometric authentication model exhibited 0% EER and 100% accuracy when tested with 25 users in sitting positions. Our future work will consider evaluating the proposed model with a larger dataset of users in multiple positions. Future work is also planned to evaluate the end-to-end security performance of the proposed model.

## Figures and Tables

**Table 1:** Notations.

Notation	Description
p${}_{i}$	Patient *i*
h${}_{i}$	Health worker *i*
GCp${}_{i}$	Gateway Layer *i*
hospCp${}_{i}$	Hospital Service Cloud Provider *i*
RhospCp${}_{i}$	Rough Service Provider *i*

**Table 2:** Experiment phase 1: model training comparison with 100 epochs.

Size of LSTM	TN	FN	FP	TP	Sensitivity	Specificity	Precision	Accuracy	F Score
2 Layers	107	0	0	72	100.00%	100.00%	100.00%	100.00%	100.00%
8 Layers	108	0	0	71	100.00%	100.00%	100.00%	100.00%	100.00%
16 Layers	108	0	0	71	100.00%	100.00%	100.00%	100.00%	100.00%
24 Layers	107	0	0	72	100.00%	100.00%	100.00%	100.00%	100.00%

**Table 3:** Testing results comparison.

Size of BiLSTM	TN	FN	FP	TP	Sensitivity	Specificity	Precision	Accuracy	F Score
2 Layers	46	0	0	30	100.00%	100.00%	100.00%	100.00%	100.00%
8 Layers	45	0	0	31	100.00%	100.00%	100.00%	100.00%	100.00%
16 Layers	45	0	0	31	100.00%	100.00%	100.00%	100.00%	100.00%
24 Layers	46	0	0	32	100.00%	100.00%	100.00%	100.00%	100.00%

**Table 4:** Biometric model comparison.

Biometric Trait	Work	Accuracy	EER	Subjects
PPG, ECG	Phase 2	100%	0%	25
PPG	[18]	100%	0%	10
ECG, PPG, GSR	S1a [42]	NA	0.05%	25
ECG	[46]	NA	0.11%	78
ECG	[47]	NA	0.01%	30

## Data Availability

Data supporting reported results can be downloaded from [43].
